# Can polymorphisms of AMH/AMHR2 affect ovarian stimulation outcomes? A systematic review and meta-analysis

**DOI:** 10.1186/s13048-020-00699-4

**Published:** 2020-09-04

**Authors:** Di Chen, Xiangyu Zhu, Jielei Wu

**Affiliations:** 1 Department of Neurology, Xiangya Hospital, Central South University, 87 Xiangya Road, Changsha, 410008 China; 2 Center for Reproductive medicine, Xiangya Hospital, Central South University, 87 Xiangya Road, Changsha, 410008 China

**Keywords:** AMH, AMHR2, Meta-analysis, Polymorphism, SNP

## Abstract

**Background:**

Previous studies have investigated the effects of anti-Müllerian hormone (AMH) and AMH type II receptor (AMHR2) polymorphisms on ovarian stimulation outcomes, but the results were inconsistent.

**Methods:**

We searched PubMed, Web of Science, Embase, and Cochrane Central Register of Controlled Trials databases for the literature used in this meta-analysis. The meta-analysis was performed with a random effects model with RevMan 5.3.5. Results were expressed as the relative risk (RR) for discrete data and the mean difference (MD) for continuous outcomes with a 95% confidence interval (CI).

**Results:**

Seven studies with 2078 participants were included. More metaphase II (MII) oocytes were retrieved in the T allele carrier of AMH (rs10407022) in the dominant model (MD: 1.20, 95% CI: 0.76 to 1.65, I^2^ = 0%, *P* < 0.00001), homozygote model (MD: 1.68, 95% CI: 0.35 to 3.01, I^2^ = 70%, *P* = 0.01) and heterogeneity model (MD: 1.20, 95% CI: 0.74 to 1.66, I^2^ = 0%, *P* < 0.00001). Oocytes retrieved from the Asian region in the TT carrier were significantly lesser than those in the GG/GT carrier in AMH (rs10407022) (MD: -1.41, 95% CI: − 1.75 to − 1.07, I^2^ = 0%). Differences in the stimulation duration, gonadotropin (Gn) dosage, and pregnancy rate were insignificant.

**Conclusions:**

Our analysis indicated that the polymorphisms of AMH/AMHR2 could influence the ovarian stimulation outcomes. Prospective studies with a larger sample size and more rigorous design are needed in the future to further confirm these findings.

## Introduction

The anti-Müllerian hormone (AMH), also known as Müllerian-inhibiting substance, belongs to the transforming growth factor-beta (TGF-β) superfamily of growth and differentiation factors [1]. AMH is synthesized by granulosa cells of preantral and small antral follicles [2], and its level strongly correlates with the size of primordial follicle pool and the number of antral follicles [3], which has made AMH an ideal marker of the ovarian reserve [4].

AMH plays a key role in the regulation of primordial follicle recruitment and cyclic selection. Through modulating the threshold of follicle-Stimulating Hormone (FSH) sensitivity, AMH could inhibit FSH-induced antral follicle growth and limit the transition of follicles from the primordial to primary stage [5, 6]. AMH exerts its specific biological function mainly through the AMH type II receptor (AMHR2), which is expressed on granulosa and theca cells [7].

Considering the potential role of AMH in affecting ovarian response to stimulation, it has been proposed that variation in the genes encoding the AMH signaling pathway may influence the ovarian response during controlled ovarian stimulation (COS). The AMH gene is located on the short arm of chromosome 19 and consists of 5 exons [8, 9]. The gene of AMHR2 is located on chromosome 12 and is comprised of 11 exons [10]. Several polymorphisms related to these two genes have been studied. The polymorphisms AMH c.146G > T, p.Ile49Ser (rs10407022) and AMHR2 -482A > G (rs2002555) have drawn the most attention. The AMH rs10407022 polymorphism rests in the promoter region. This polymorphism leads to the replacement of serine from isoleucine in the position 49 of AMH protein, and it can affect AMH bioactivity [11]. The AMHR2 rs2002555 polymorphism is located in the non-coding region of the promoter, and it can affect the transcription process of AMHR2. Several studies have focused on these two polymorphisms and have suggested that these two polymorphisms are associated with elevated follicular phase estradiol levels in normo-ovulatory women [12], unexplained infertility [13], follicle number, and androgen levels in polycystic ovary syndrome (PCOS) [14]. Some studies have also investigated the effects of these two polymorphisms during COS in assisted reproduction technology (ART) treatment [15–21]. However, the results of these studies were inconsistent. A meta-analysis of the polymorphism AMH (rs10407022) has been published [22]. However, this study only explored the association between AMH polymorphisms and reproductive outcomes in the Caucasian population. Since then, several new studies on SNPs of the AMH/AMHR2 pathway have been published. Considering this, we feel that it is clinically important to conduct a meta-analysis to comprehensively evaluate the role of AMH (rs10407022) and AMHR2 (rs2002555) in the ovarian response and the outcomes of in vitro fertilization (IVF) during the process of ovarian stimulation.

## Materials and methods

We followed the Preferred Reporting Items for Systematic Reviews and Meta-Analyses (PRISMA) reporting guidelines to design and report this systematic review and meta-analysis [23].

### Search strategy

Studies were searched from PubMed, Web of Science, Embase, and Cochrane Central Register of Controlled Trials databases published without language restriction from inception to December 2019. The search strategies used a combination of terms “polymorphism,” “pharmacogenetics,” “AMH,” “AMHR,” and “controlled ovarian stimulation.” The detailed search strategies are provided in the supplemental material (Appendix 1). Reference lists of relevant reviews and articles were manually searched.

### Eligibility criteria

The criteria of the inclusion of studies were as follows: (1) participants underwent IVF/ intracytoplasmic sperm injection (ICSI); (2) single nucleotide polymorphisms (SNPs) of AMH and AMHR2 were detected in some or all of the participants; (3) COS outcomes based on the gene polymorphisms were available.

### Study selection

After removing duplicates, titles and abstracts were screened by two individual reviewers. Disagreements were discussed and resolved by consensus. Only trials published in peer-reviewed journals were included. Case reports, case series, conference abstracts, reviews, editorials, and gray literature were excluded.

### Data extraction

Data were extracted independently from all eligible articles by two reviewers, and they included the first author, publication year, region, SNPs reported, sample size, treatment protocol, study design, and outcomes. If the median and percentile values rather than the mean and standard deviation (SD) were provided, the data were converted to mean and SD through the method described elsewhere [24]. Two subgroups (e.g. AA vs. AB, AA vs. BB) were combined into one group (e.g. AA vs. BB/AB) by referring to the method described in the Cochrane Handbook for Systematic Reviews of Interventions if necessary [25]. Consensus was reached to resolve the discrepancies.

### Quality assessment

Two independent reviewers assessed the quality of included studies by the Newcastle-Ottawa scale (NOS) scores. The judgment of NOS scores was based on the following three domains: selection of the study group, comparability between groups, and ascertainment of exposed/not exposed cohorts [26].

### Outcomes of interest

The primary outcome was defined as the number of retrieved oocytes. The secondary outcomes included stimulation duration, Gn dosage, the number of metaphase II (MII) oocytes, and pregnancy rate. Pregnancy was defined as at least one gestational sac with a fetal heart activity under ultrasonographic visualization.

### Statistical analysis

The meta-analysis was performed with the Review Manager software (Revman), version 5.3.5. Relative risk (RR) was used for categorical data. Mean difference (MD) was used for continuous data. All of the outcomes were calculated with 95% confidence intervals (CIs). Four genetic models were used in this study (dominant model: AA vs Aa/aa; homozygote model: AA vs aa; heterozygote model AA vs Aa; and recessive model: aa vs AA/Aa). A random effects model was used as the clinical heterogeneity existed among studies. We evaluated the heterogeneity between studies using Cochran’s Q statistic with associated *P*-value [27]. The degree of heterogeneity was quantified by measuring I^2^. I^2^ > 50% and *P* < 0.05 indicated substantial heterogeneity. Subgroup analysis was performed to explore the source of heterogeneity. Sensitivity analysis by sequentially removing an individual study was also conducted to investigate the source of heterogeneity and the stability of the results. Statistical significance was set at *P* < 0.05.

## Results

### Identification of studies and quality assessment

**Fig. 1:**
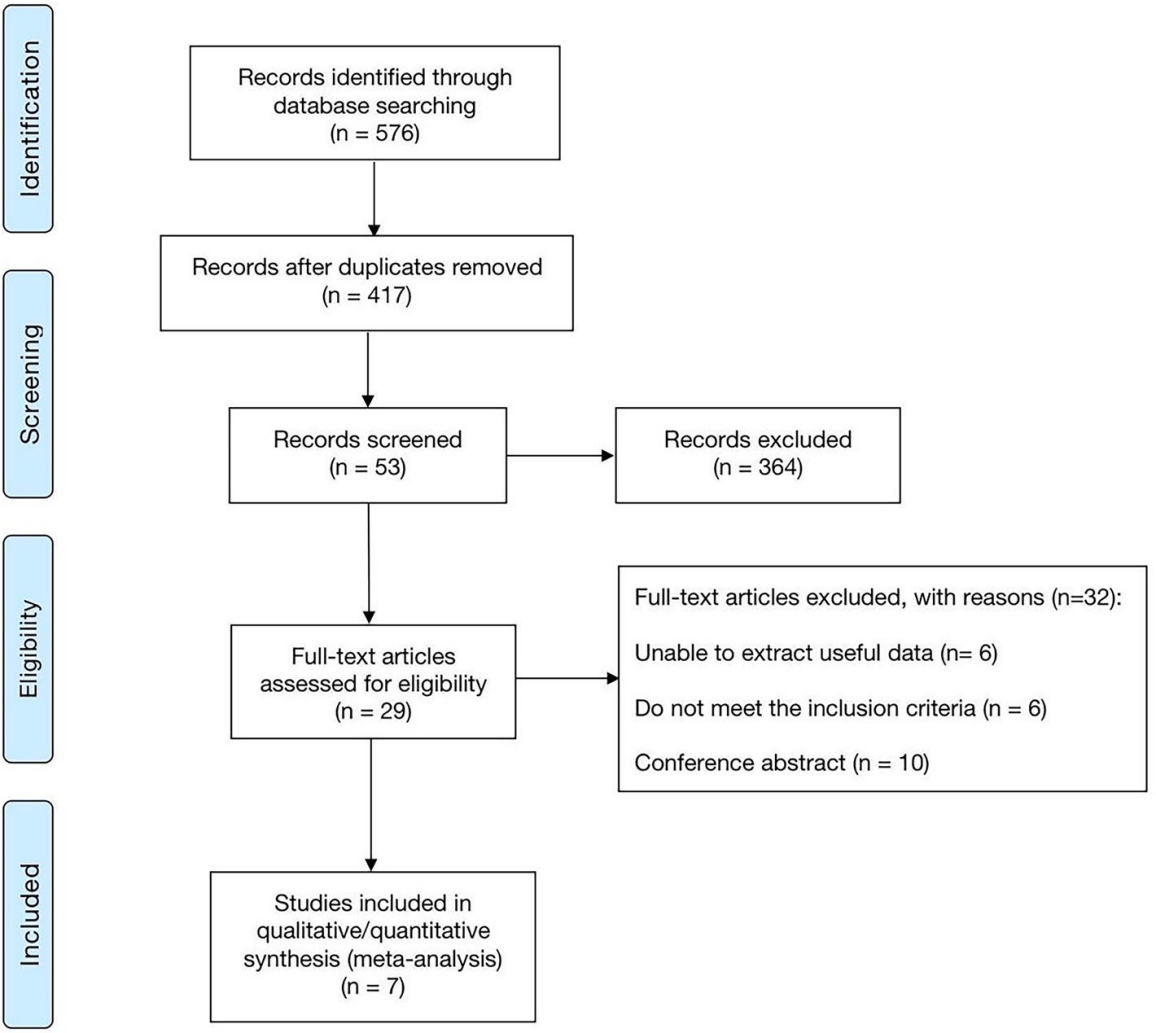
PRISMA flow chart of literature retrieval and study selection

A flow chart of study selection is shown in Fig. 1. A total of seven studies were included in this systematic review and meta-analysis. The characteristics of the included studies are presented in Table 1. All studies were published between 2015 and 2019. Among these trials, two were performed in China [20, 21], four studies originated from European countries [15–18], and one study was from Brazil [19]. The sample size ranged from 122 to 635 with 2078 participants in total. The detailed assessment of bias within studies is shown in Table S1 and the NOS score of the studies varied between 6 and 7.

**Table 1:** The Characteristics and Newcastle–Ottawa scale score of studies included in the meta-analysis

Study	Country/Ethnicity	SNPs evaluated	No. of patients	Mean age ± SD	Protocol	Study design	Genotyping	HWE	Outcomes*	NOS scores
Lledó, 2019 [18]	Spain/Caucasian	AMH/ AMHR2	124	23.9 ± 3.5	GnRH-A	prospective	PCR	Yes	1.2.3	6
Wu, 2019 [21]	China/Asian	AMH/AMHR2	635	NA	GnRHa	prospective	PCR	Yes	1.2.3.4.7	6
Lazaros, 2016 [17]	Greece/Caucasian	AMHR2	300	28–38	GnRHa	prospective	PCR	Yes	3. 4.	7
Cerra, 2016 [15]	UK/multiple	AMH/AMHR2	603	NA	GnRHa/GnRH-A	prospective	TaqMan	No metion	2. 3. 5. 8	6
Peluso, 2015 [19]	Brazil	AMH/AMHR2	186	32.5 ± 3.5	GnRH-A	prospective	TaqMan	Yes	3. 6. 7. 8.	6
Wang, 2015 [20]	China/Asian	AMH	122	NA	GnRHa	retrospective	PCR	Yes	3	7
Karagiorga, 2015 [16]	Greece	AMH/AMHR2	151	36 ± 5	GnRHa/GnRH-A	prospective	PCR	Yes	1. 2. 3. 4.	7

*Outcomes: 1. stimulation duration; 2. Gn dosage; 3. No. of oocytes retrieved; 4. clinical pregnancy rate; 5. live birth; 6. AMH level; 7. No. of MII oocytes; 8. No. of embryo. *HWE* Hardy-Weinberg equilibrium

### The summary of results

**Fig. 2:**
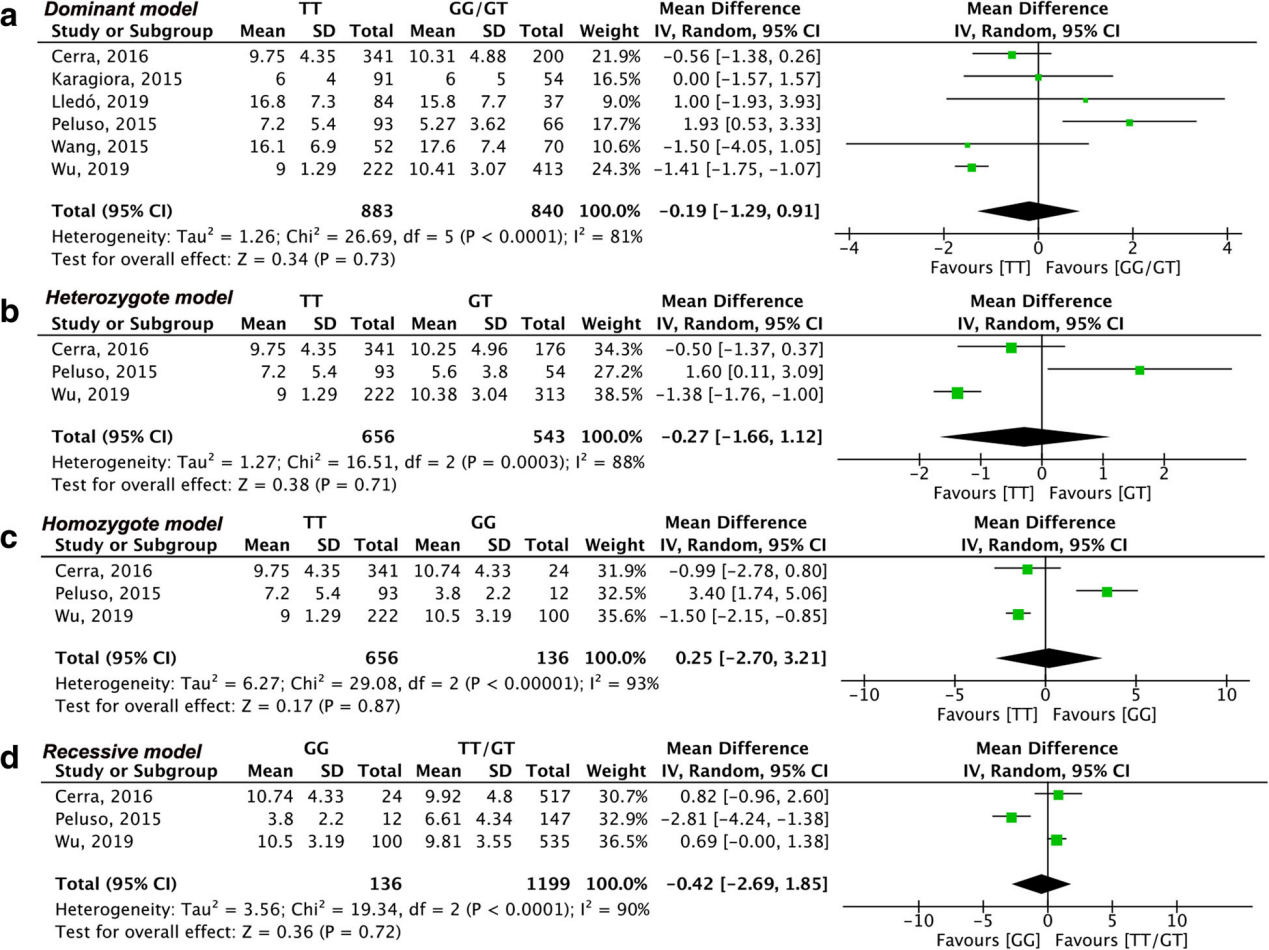
Forest plots of differences among AMH (rs10407022) genotype carriers regarding the number of oocytes retrieved. **(a)** dominant model, (**b**) heterozygote model, (**c**) homozygote model, (**d**) recessive model

**Fig. 3:**
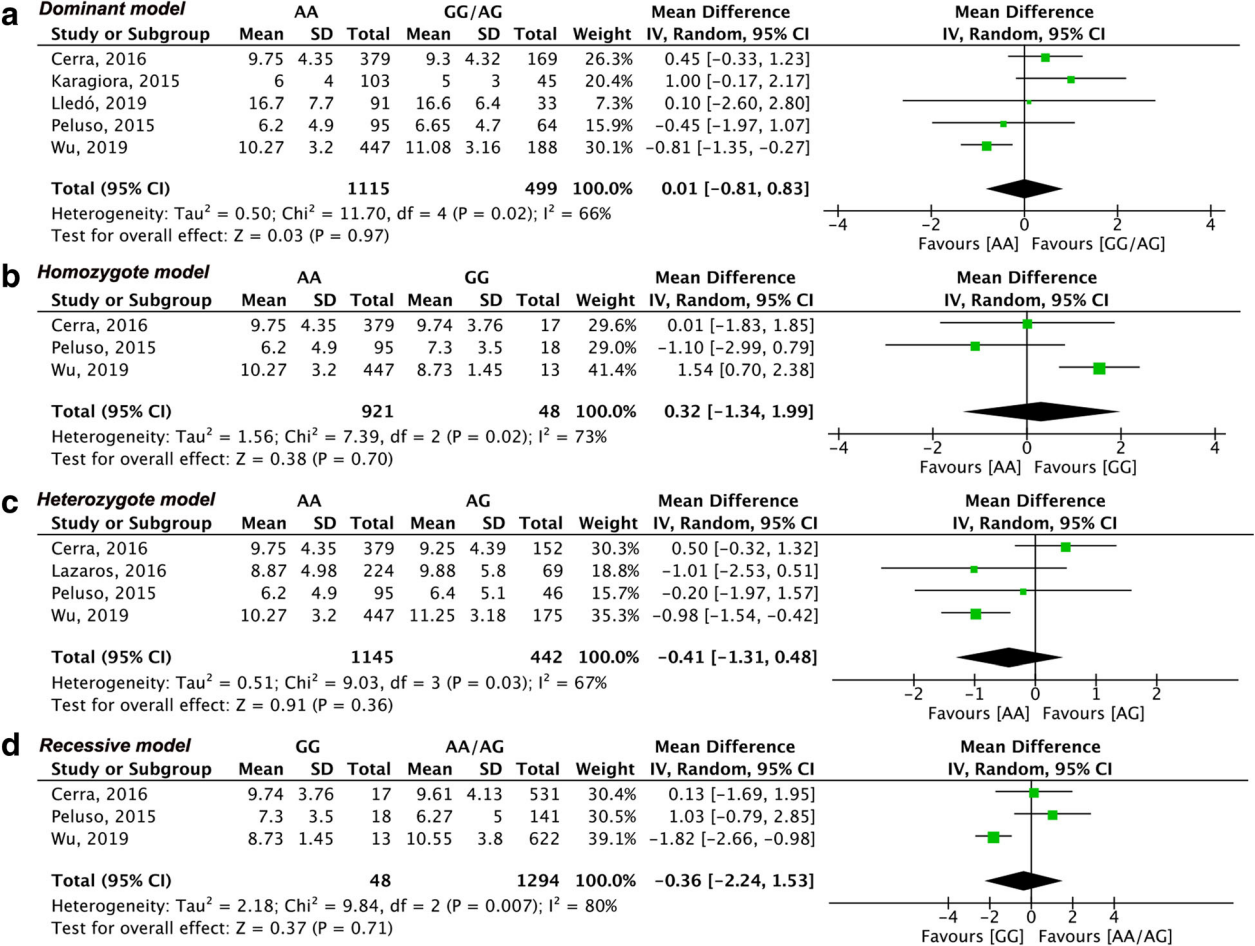
Forest plots of differences among AMHR2 (rs2002555) genotype carriers regarding the number of oocytes retrieved. **(a)** dominant model, **(b)** homozygote model, **(c)** heterozygote model, **(d)** recessive model

**Fig. 4:**
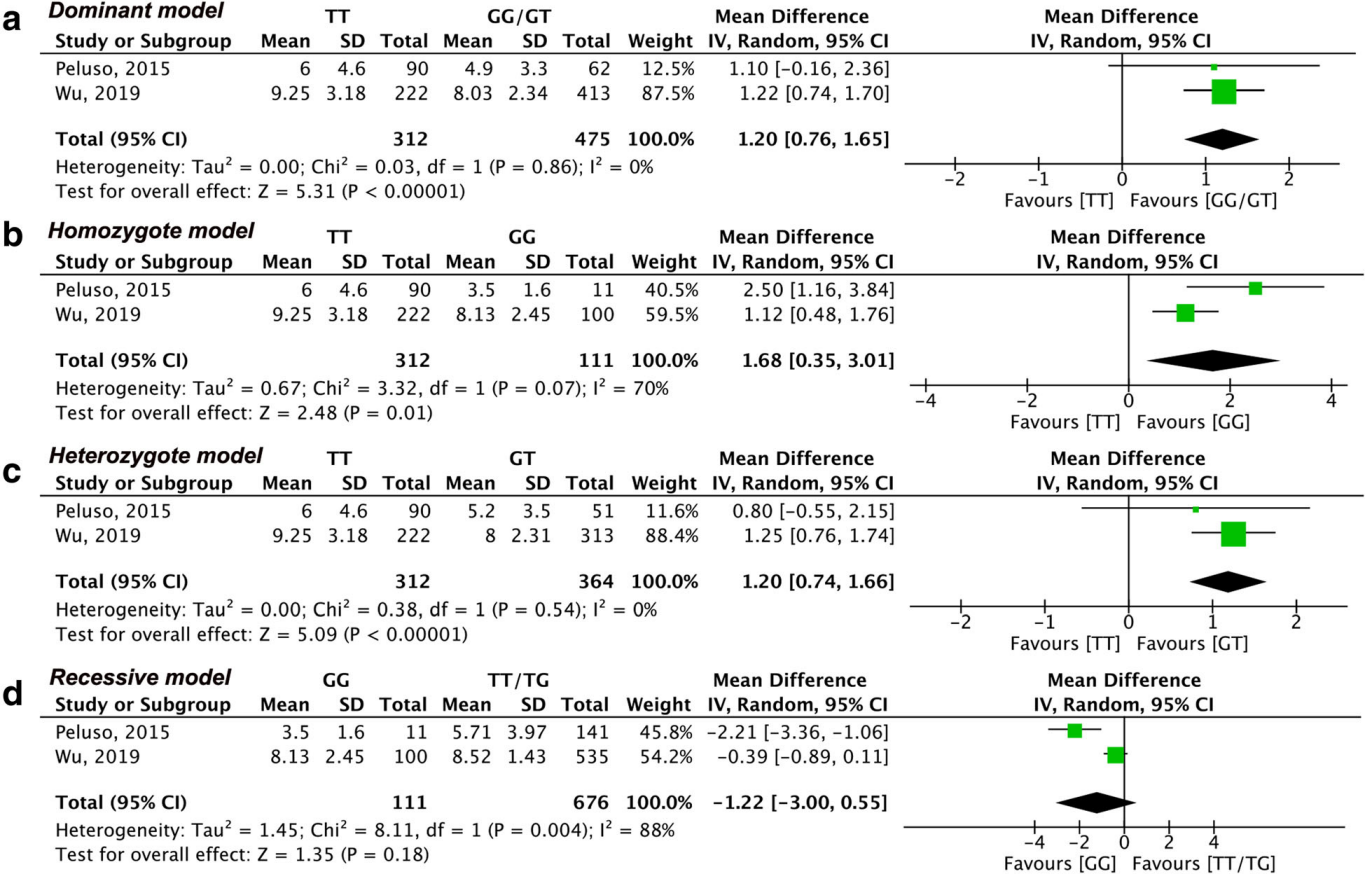
Forest plots of differences among AMH (rs10407022) genotype carriers regarding the number of MII oocytes. **(a)** dominant model, (**b**) homozygote model, (**c**) heterozygote mode, (**d**) recessive model

**Fig. 5:**
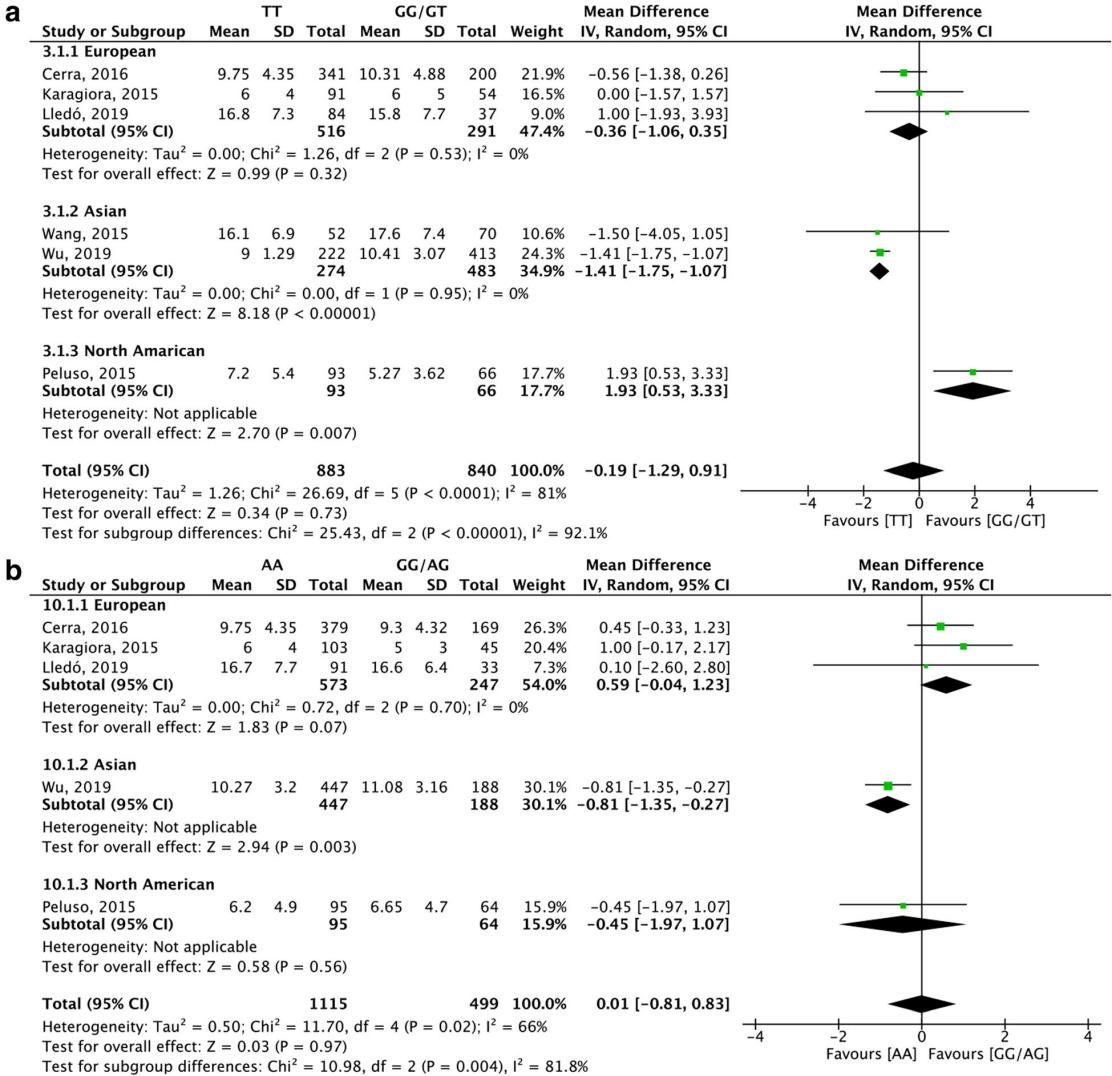
Forest plots of subgroup analysis for the number of oocytes retrieved. **(a)** AMH (rs10407022), (**b**) AMHR2 (rs2002555)

The result of the meta-analysis of each outcome based on genotype distribution is summarized in Table 2. Due to the limited number of eligible studies included, we only estimated the number of oocytes retrieved in the dominant model through region-based subgroup analysis. Sensitivity analysis was conducted when included studies were more than three. The summary of sensitivity analysis is shown in Table 3. Meanwhile for better illustration, we only showed the primary outcome or results with significance in Figs. 2, 3, 4 and 5 and the results without significance are presented as supplementary data.

**Table 2:** Pooled effect estimates AMH and AMHR2 effect on ovarian stimulation outcomes

Variant	Genetic model	Parameter	study(N)	sample size (N)	Overall effect			
					MD	95% CI	I^2^(%)	P
AMH (rs10407022)	Dominant (TT versus GG/GT)	Gn dosage	4	1448	11.75	−38.70, 62.20	17	0.65
		stimulation duration	3	907	0.07	−0.16, 0.31	37	0.53
		oocytes retrieved	6	1723	−0.19	−1.29, 0.91	81	0.73
		MII oocytes	2	787	**1.20**	**0.76, 1.65**	**0**	**<0.00001**
	Homozygote (TT versus GG)	Gn dosage	2	687	215.22	−142.04, 572.47	86	0.24
		oocytes retrieved	3	792	0.25	−2.70, 3.21	93	0.87
		MII oocytes	2	423	**1.68**	**0.35, 3.01**	**70**	**0.01**
	Heterozygote (TT versus GT)	Gn dosage	2	1052	−35.47	−74.68, 3.74	0	0.08
		oocytes retrieved	3	1199	−0.27	−1.66, 1.12	88	0.71
		MII oocytes	2	676	**1.2**	**0.74, 1.66**	**0**	**<0.00001**
	Recessive (GG versus TT/GT)	Gn dosage	2	1335	−0.42	−2.69, 1.85	90	0.72
		oocytes retrieved	2	679	−0.42	−2.69, 1.85	90	0.72
		MII oocytes	2	787	−1.22	−3.00, 0.55	88	0.18
AMHR2 (rs2002555)	Dominant (AA versus GG/AG)	Gn dosage	4	1230	−13.25	− 109.45, 82.95	63	0.79
		Stimulation duration	3	907	2.81	−4.51, 10.13	100	0.45
		oocytes retrieved	5	1614	0.01	−0.81, 0.83	66	0.97
		MII oocytes	2	787	−0.02	− 0.52, 0.49	4	0.95
Variant	Genetic model	Parameter	study(N)	sample size (N)	Overall effect			
					MD	95% CI	I^2^(%)	P
AMHR2 (rs2002555)	Homozygote (AA versus GG)	Gn dosage	2	631	− 150.35	− 319.02, 18.32	0	0.08
		oocytes retrieved	3	969	0.32	−1.34, 1.99	73	0.70
		MII oocytes	2	571	0.22	−1.98, 2.42	82	0.85
	Heterozygote (AA versus AG)	Gn dosage	2	928	52.37	−73.00, 177.75	74	0.41
		oocytes retrieved	4	1587	−0.41	−1.31, 0.48	67	0.36
		MII oocytes	2	756	−0.05	− 0.55, 0.45	0	0.84
	Recessive (GG versus AA/AG)	Gn dosage	2	958	147.15	−19.30, 313.60	0	0.08
		oocytes retrieved	3	1342	−0.36	−2.24, 1.53	80	0.71
		MII oocytes	2	787	−0.27	−2.27, 1.73	80	0.79

MD: mean difference; CI: confidence interval; P: *p* value for association with significance set at < 0.05; I^2^ values as measure of heterogeneity are considered low (< 50%), moderate (51–74%) or high (> 75%); values in **bold** indicate significant associations;

**Table 3:** Sensitivity analysis

Outcome	Polymorphism	Comparison	All studies	MD (95%CI)	I^**2**^	Sensitivity analysis			Results
			No. Studies/ participant			No. Studies/ participant	MD 95%CI	I^**2**^	
No. of oocytes retrieved	*AMH (rs10407022)*	TT versus GG/GT	6/1739	−0.19 (−1.29, 0.91)	81%	^a^ 5/1580	−0.82 (− 1.55, − 0.08)	52%	Became significant
		TT versus GG	3/792	0.25 (−2.70, 3.21)	93%	^a^ 2/687	−1.44 (−2.05, −0.83)	0%	Became significant
		TT versus GT	3/1199	−0.27 (−1.66, 1.12)	88%	^a^ 2/1052	−1.03 (− 1.87, − 0.19)	70%	Became significant
		GG versus TT/GT	3/1335	−0.42 (− 0.2.69, 1.85)	90%	^a^ 2/1176	0.71 (0.06, 1.53)	0%	Became significant
	*AMHR (rs2002555)*	AA versus GG/AG	5/1614	0.01 (−0.81,0.83)	66%	^b^ 4/979	0.44 (−0.15, 1.02)	0%	Not affected
		AA versus GG	3/969	0.32 (−1.34, 1.99)	73%	^b^ 2/509	−0.53 (− 1.85, 0.79)	0%	Not affected
		AA versus AG	4/1587	−0.41 (−1.31, 0.48)	67%	^c^ 3/1056	−0.92 (−1.42, − 0.42)	0%	Became significant
		GG versus AA/AG	3/1342	−0.36 (−2.24, 1.53)	80%	^b^ 2/707	0.58 (−0.70, 1.87)	0%	Not affected
Gn dosage	*AMHR (rs2002555)*	AA versus GG/AG	4/1230	−13.25 (−58.41, 32.47)	63%	^c^ 3/682	−53.36 (−146.58, 39.86)	44%	Not affected
Stimulation duration	*AMHR (rs2002555)*	AA versus GG/AG	3/907	2.81 (−4.51, 10.13)	75%	^b^ 2/270	−0.27 (−0.86, 0.31)	66%	Not affected

^a^ Sensitivity analysis excluding Peluso study

^b^ Sensitivity analysis excluding Wu study

^c^Sensitivity analysis excluding Cerra study

*AMH* anti-Müllerian hormone, *AMHR* AMH receptor, *Gn dosage* gonadotropin consumption, *MD* weighted mean difference, *No.* number

### Primary outcome

#### Oocytes retrieved

Six studies [15, 16, 18–21] with a total of 1729 participants reported the number of oocytes retrieved in relation to the distribution of AMH (rs10407022) genotypes. The difference in oocytes retrieved was insignificant in the dominant model (MD: -0.19, 95% CI: − 1.29 to 0.91, I^2^ = 81%, Fig. 2a). The number of retrieved oocytes in TT homozygotes was close to that in GT heterozygotes (MD: -0.27, 95% CI: − 1.66 to 1.12, I^2^ = 88%, Fig. 2b), and GG homozygotes (MD: 0.25, 95% CI: − 2.70 to 3.21, I^2^ = 93%, Fig. 2c). The number of oocytes retrieved was not significantly different in the recessive model (MD: -0.42, 95% CI: − 2.69 to 1.85, I^2^ = 90%, Fig. 2d). Subgroup analysis according to the region in the dominant model showed significant statistical association in the Asian region (MD: -1.41, 95% CI: − 1.75 to − 1.07, I^2^ = 0%, Fig. 5a). Furthermore, the heterogeneity in the European region became acceptable, although without a significant change (MD: -0.36, 95% CI: − 1.06 to 0.35, I^2^ = 0%, Fig. 5a). The leave-one out sensitivity analysis indicated that Peluso’s [19] study was the main source of heterogeneity in all genetic models, and the overall effect even changed into being significant after omitting Peluso’s [19] study (Table 3).

Six studies [15–19, 21] including 1907 participants reported the number of retrieved oocytes regarding the distribution of AMHR2 (rs2002555) genotypes. There was no difference in the dominant model (MD: 0.01, 95% CI: − 0.81 to 0.83, I^2^ = 66%, Fig. 3a). Similar results were also observed in other genetic models. The number of oocytes retrieved from AA homozygotes was comparable to that retrieved from GG homozygotes (MD: 0.32, 95% CI: − 1.34 to 1.99, I^2^ = 73%, Fig. 3b) and AG heterozygotes (MD: -0.41, 95% CI: − 1.31 to 0.48, I^2^ = 67%, Fig. 3c). No significant difference in the number of retrieved oocytes was observed in the recessive model (MD: -0.36, 95% CI: − 2.24 to 1.53, I^2^ = 80%, Fig. 3d). Subgroup analysis by region in the dominant model showed a similar result, but the heterogeneity became acceptable in the European region, besides only one study was included in the other two regions (Fig. 5b). According to the sensitivity analysis, the studies by Wu [21] and Cerra [15] were the potential sources of heterogeneity. Furthermore, the pooled results of retrieved oocytes in the heterozygote model became statistically significant after removing the study by Cerra [15] (Table 3).

### Secondary outcomes

#### Gn dosage

Four studies [15, 16, 18, 21], with 1448 participants, evaluated the Gn dosage regarding the genotype distribution of AMH (rs10407022). The differences in the Gn dosage in all the models (Supplementary Fig. 1C) were insignificant.

With respect to the genotype distribution of AMHR2 (rs2002555), these four studies with a total of 1230 women reported the Gn dosage. No significant differences in the Gn dosage were found in all of the AMHR2 genetic models. Our sensitivity analysis indicated that Cerra study was the potential source of heterogeneity in the dominant model (Table 3).

#### Stimulation duration

Three studies [16, 18, 21] with a total of 907 patients reported the stimulation duration in terms of the distribution of the AMH (rs10407022) genotype. No difference was observed between the TT carriers versus GT/GG carriers (MD: 0.07, 95% CI: − 0.16 to 0.31, I^2^ = 37%; Supplementary Fig. 3A).

These three studies also evaluated the duration of stimulation in relation to AMHR2 (rs2002555) genotype distribution. Similarly, the stimulation duration did not differ among AA homozygotes and GG/AG heterozygotes (MD: 2.81, 95% CI: − 4.51 to 10.13, I^2^ = 100%; Supplementary Fig. 3B).

#### Number of MII oocytes

Two studies [19, 21] including a total of 787 participants assessed the number of MII oocytes in relation to the distribution of both AMH (rs10407022) and AMHR2 (rs2002555) genotypes. With respect to the distribution of the AMH (rs10407022) genotype, there was a significant difference in the number of MII oocytes in the dominant model (MD: 1.20, 95% CI: 0.76 to 1.65, I^2^ = 0%, *P* < 0.00001, Fig. 4a). Besides the number of MII oocytes in the TT homozygotes was greater than that in the GG homozygotes (MD: 1.68, 95% CI: 0.35 to 3.01, I^2^ = 70%, *P* = 0.01, Fig. 4b) and GT heterozygotes (MD: 1.20, 95% CI: 0.74 to 1.66, I^2^ = 0%, *\* 0.00001, Fig. 4c). An insignificant difference in MII oocytes was observed in the recessive model (MD: -1.22, 95% CI: − 3.00 to 0.55, I^2^ = 88%, Fig. 4d).

Regarding the AMHR2 (rs2002555) genotype distribution, the number of MII oocytes was not statistically different in all genetic models (Supplementary Fig. 4).

#### Pregnancy rate

Two studies [16, 21] with 786 participants evaluated the pregnancy rate in the distribution of the AMH (rs10407022) genotype. An insignificant difference was observed between TT carriers and GG/GT carriers (RR: 1.12, 95% CI: 0.96 to 1.30, I^2^ = 0%, Supplementary Fig. 5A).

Three studies [16, 17, 21] with 1076 participants reported the pregnancy rate regarding the AMHR2 (rs2002555) genotype distribution. The pregnancy rate of AA homozygotes was close to that of AG heterozygotes (RR: 0.97, 95% CI: 0.83 to 1.13, I^2^ = 0%, Fig. S5B); similar results were found in the dominant model (RR: 0.97, 95% CI: 0.82 to 1.14, I^2^ = 0%, Supplementary Fig. 5C).

## Discussion

There is growing evidence supporting that SNP may contribute to the differences in complex characteristics between individuals. Previous studies on the AMH/AMHR2 signaling pathway have revealed that polymorphisms of AMH and AMHR2 may associate with the ovarian response. A meta-analysis by Pabalan et al. [22] investigated the association of AMH rs10407022 and AMHRII rs2002555 with reproductive outcomes and PCOS. They found no evidence of significant associations of the two polymorphisms with reproductive outcomes and PCOS, and they also found that AMH rs10407022 could increase the risk of PCOS up to 1.5-fold in Caucasians.

Therefore, we focused on the association between the AMH/AMHR2 gene polymorphisms and ovarian stimulation outcomes, and then we provided a more comprehensive evaluation of the outcomes of assisted reproductive technology (ART)herapy.

The primary outcome of our study was the number of retrieved oocytes. It is closely related to the success rate of ART therapy-the more the number of oocytes retrieved, the higher the cumulative delivery rate [28]. In this study, AMH (rs10407022) polymorphism had no association with the number of oocytes retrieved, even though in the subgroup analysis based on the regions, the number of oocytes retrieved in TT homozygotes was significantly lower than that retrieved in GG/GT carriers in the Asian region. In addition, when the Peluso [19] study was omitted in these four genetic models, all of the results turned significant, and the T allele carriers had obviously fewer retrieved oocytes than the G allele carriers. The AMHR2 (rs2002555) seemed to have no effect on the oocytes retrieved, although the results became significant after removing the study by Cerra [15] in the heterozygote model.

In the sensitivity analysis, we found that eliminating the studies by Peluso [19] or Cerra [15] could change the significance of the results of the oocytes retrieved. One potential reason could be the region or ethnicity difference among the studies since different ethnicities may have different allelic frequencies [19, 21]. Another reason may be different genotyping techniques used in the study because only Cerra [15] and Peluso [19] used TaqMan and other researchers used PCR. The differences in the age of the participants and treatment protocols could also have contributed to the high heterogeneity.

The previous meta-analysis only reported that AMH (rs10407022) reduced the risk in reproductive outcomes and increased the risk of PCOS among Caucasian population. In our study, in the European region, we did not find a significant association between AMH (rs10407022) and the number of oocytes retrieved. Indeed, we found that the number of oocytes was smaller in TT carriers in the dominant model in the Asian region. Different results may have arisen from different populations focused on. The previous meta-analysis mainly targeted the PCOS patients, while in our study, we included not only the PCOS patients but also the healthy patients. Among the seven studies included in this analysis, three studies [17, 19, 20] mentioned that they excluded participants with PCOS, Lazaros [16] only reported the included participants without the sign of hyperandrogenism, while the other two studies [15, 18] also included the PCOS population. Different inclusion and exclusion criteria may have led to this difference and high heterogeneity. However, in the subgroup analysis on the PCOS population, we found no significant difference between the two subgroups (data not shown). Therefore, excluding PCOS patients or not has a limited effect on the result and is not the main source of heterogeneity.

In the analysis of MII oocytes, we found that even though the T allele carriers of AMH rs10407022 had significantly fewer oocytes and they tended to have more MII oocytes. Some studies have found that high follicular FSH levels could interfere with the meiotic division and increase aneuploidy rates of oocytes during IVF treatment [29, 30]. Consistent with these findings, we found that the T allele carriers of AMH rs10407022 polymorphism tended to have lower basal FSH (data not shown). Based on this finding, we speculated that the function of AMH protein translated from G mutation may somehow be impaired; it could lead to high FSH in the circulation and follicle liquid, and then disturb the maturation of oocytes and eventually cause less MII oocytes. With respect to the Gn dosage, stimulation duration, and the pregnancy rate, our analysis showed that these two polymorphisms barely affected these outcomes.

In conclusion, we think that SNPs of the AMH/AMHR2 pathway, especially AMH rs10407022, could affect the number of retrieved oocytes and MII oocytes, but the specific mechanism needs further exploration.

This study indicated that the polymorphisms of AMH/AMHR2 could affect the outcomes of COS; however several limitations need to be addressed. First, inherent heterogeneity, such as the baseline characteristics of patients, ovarian stimulation protocol, and study design, existed among studies. Second, as all the included studies were observational, some unknown confounders could not be excluded, which could have caused extra bias in our estimates. Third, the number of the studies and the sample size of the included studies in our analysis were relatively small. Ideally, the COH outcomes should have been corrected with the AMH level. However, we were unable to do so due to the lack of AMH level. Moreover, a region-based subgroup analysis was limited in explaining the source of heterogeneity, as the number of studies from North American and Asian regions were relatively small.

## Conclusion

Overall, our study indicated that SNPs of the AMH/AMHR2 signaling pathway could influence the results of COS. Fewer oocytes but more MII oocytes were retrieved in T allele carriers of AMH (rs10407022) polymorphism. However, trials involving pharmacogenomic approaches on this topic and prospective studies with larger sample sizes as well as better study designs are needed in the future.

## Supplementary information

**Additional file 1 Supplementary Fig. 1.** Forest plots of differences among AMH (rs10407022) genotype carriers regarding the Gn dosage. (A) dominant model, (B) homozygote model, (C) heterozygote model, (D) recessive model.

**Additional file 2 Supplementary Fig. 2.** Forest plots of differences among AMHR2 (rs2002555) genotype carriers regarding the Gn dosage. (A) dominant model, (B) homozygote model, (C) heterozygote model, (D) recessive model.

**Additional file 3 Supplementary Fig. 3.** Forest plots of differences among AMH (rs10407022) and AMHR2 (rs2002555) genotype carriers regarding the stimulation duration. (A) dominant model of AMH (rs10407022), (B) dominant model of AMHR2 (rs2002555) dominant model.

**Additional file 4 Supplementary Fig. 4.** Forest plots of differences among AMHR2 (rs2002555) genotype carriers regarding the MII oocytes. (A) dominant model, (B) homozygote model, (C) heterozygote model, (D) recessive model.

**Additional file 5 Supplementary Fig. 5.** Forest plots of differences among AMH (rs10407022) and AMHR2 (rs2002555) genotype carriers regarding the pregnancy rate. (A) dominant model of AMH (rs10407022), (B) heterozygote model of AMHR2 (rs2002555), (C) dominant model of AMHR2 (rs2002555).

**Additional file 6.**

## Data Availability

The datasets used during the current study are available from the corresponding author on reasonable request.
