# Supplementary figures and images for: Can polymorphisms of AMH/AMHR2 affect ovarian stimulation outcomes? A systematic review and meta-analysis
Source: J Ovarian Res. 2020 Sep 4;13:103. doi: 10.1186/s13048-020-00699-4 (PMC7487641; doi:10.1186/s13048-020-00699-4)

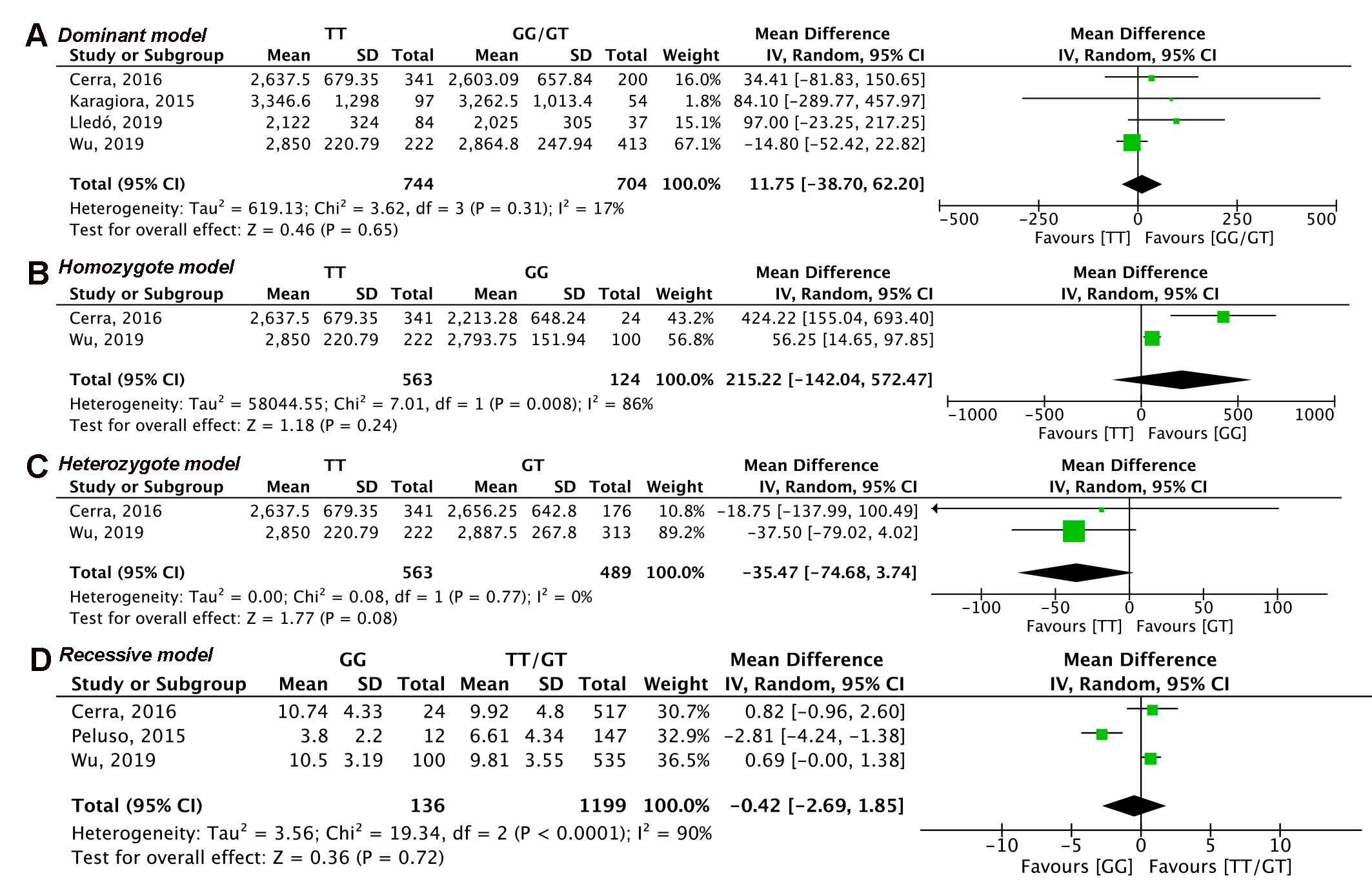

Supplement: Supplementary file 1 — Additional file 1 Supplementary Fig. 1. Forest plots of differences among AMH (rs10407022) genotype carriers regarding the Gn dosage. (A) dominant model, (B) homozygote model, (C) heterozygote model, (D) recessive model. [file 13048_2020_699_MOESM1_ESM.tif]

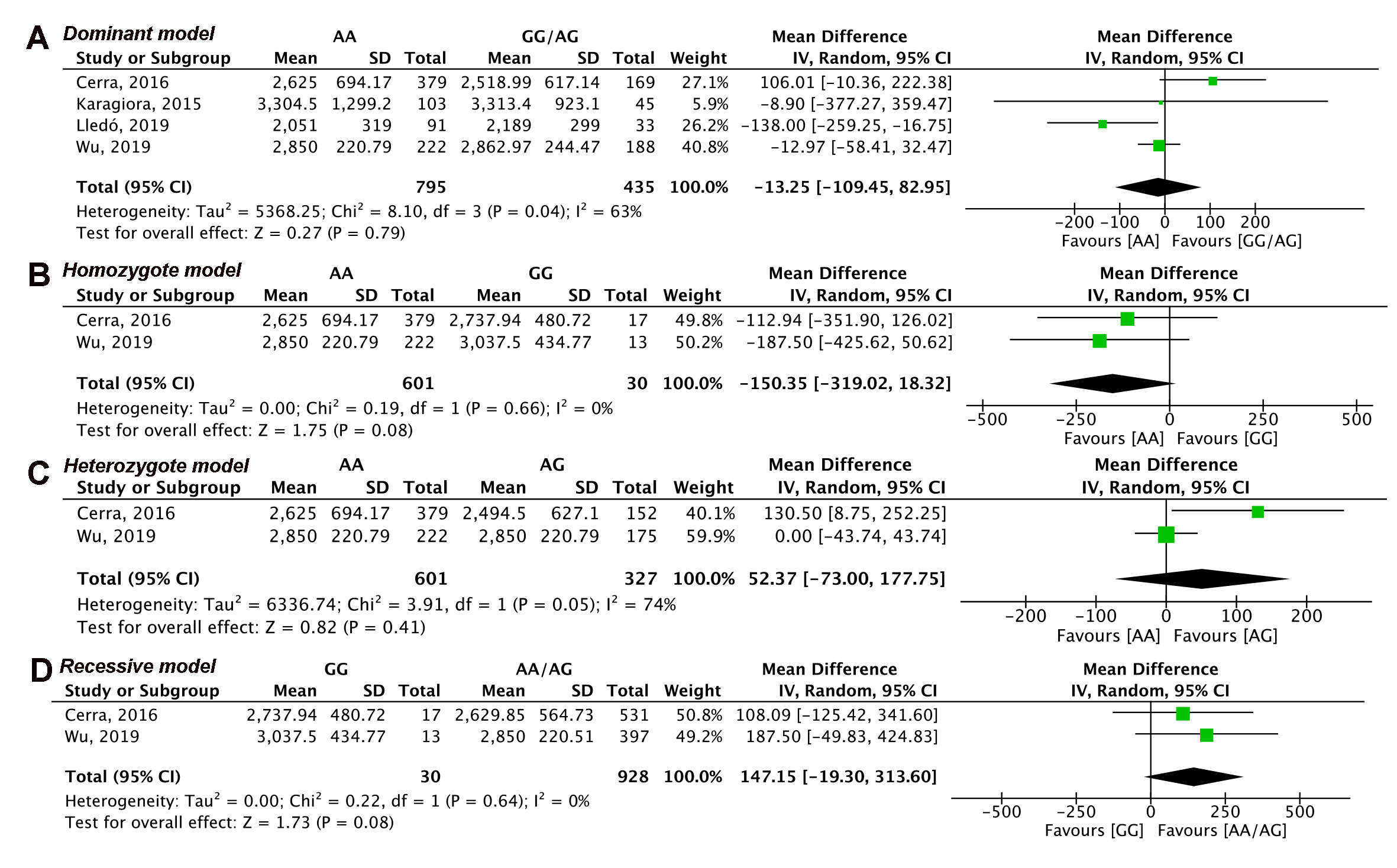

Supplement: Supplementary file 2 — Additional file 2 Supplementary Fig. 2. Forest plots of differences among AMHR2 (rs2002555) genotype carriers regarding the Gn dosage. (A) dominant model, (B) homozygote model, (C) heterozygote model, (D) recessive model. [file 13048_2020_699_MOESM2_ESM.tif]

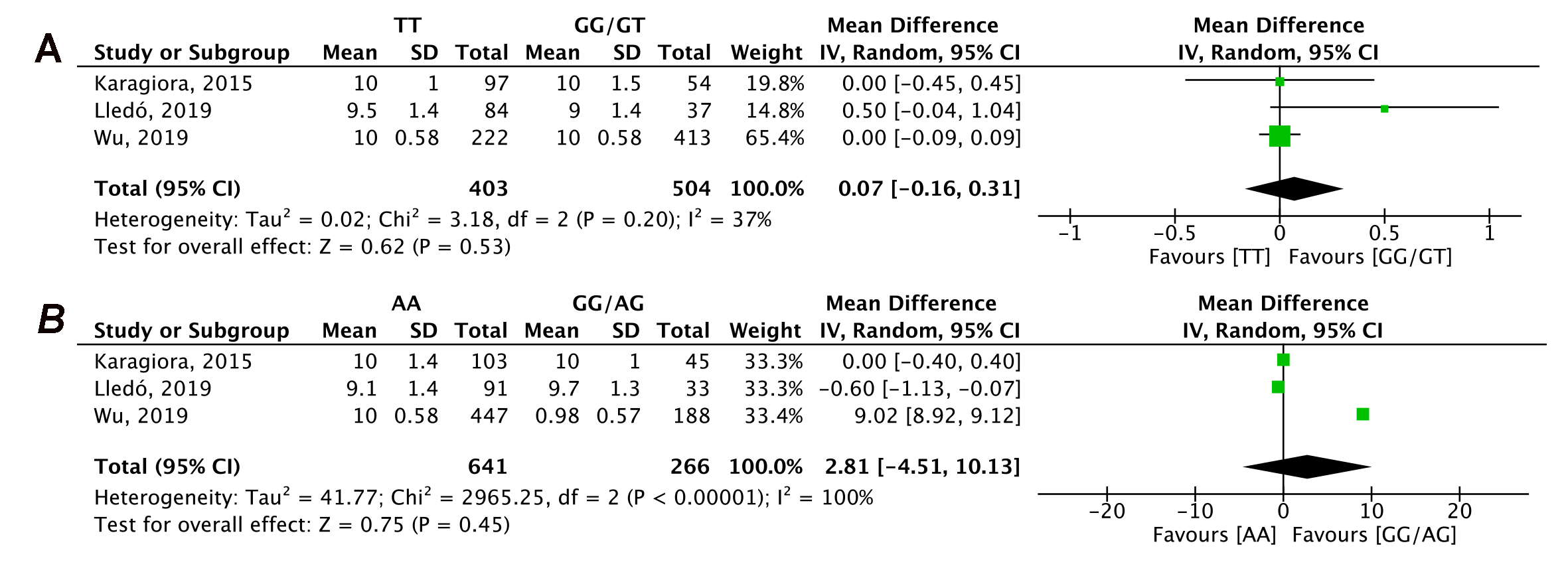

Supplement: Supplementary file 3 — Additional file 3 Supplementary Fig. 3. Forest plots of differences among AMH (rs10407022) and AMHR2 (rs2002555) genotype carriers regarding the stimulation duration. (A) dominant model of AMH (rs10407022), (B) dominant model of AMHR2 (rs2002555) dominant model. [file 13048_2020_699_MOESM3_ESM.tif]

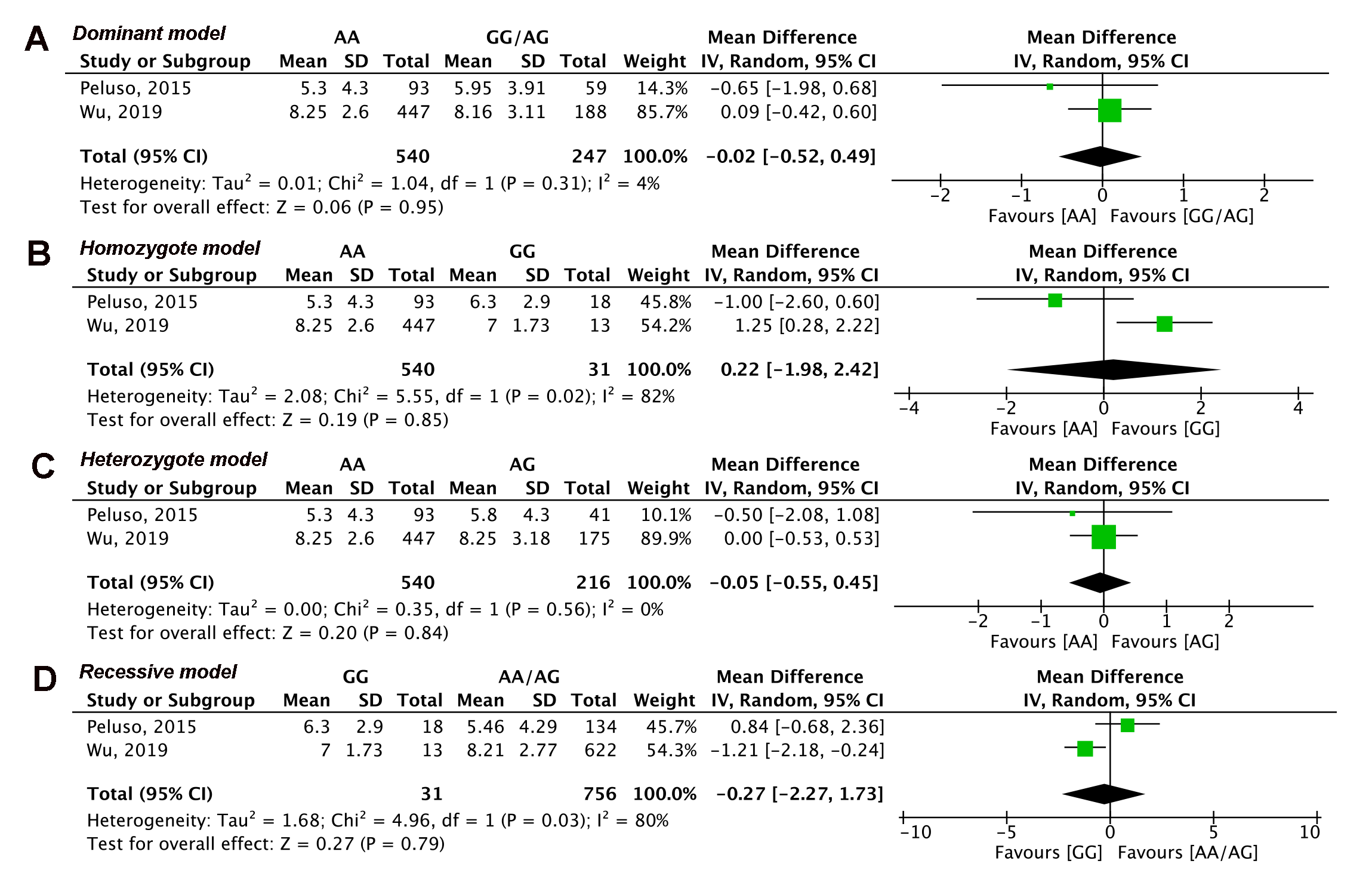

Supplement: Supplementary file 4 — Additional file 4 Supplementary Fig. 4. Forest plots of differences among AMHR2 (rs2002555) genotype carriers regarding the MII oocytes. (A) dominant model, (B) homozygote model, (C) heterozygote model, (D) recessive model. [file 13048_2020_699_MOESM4_ESM.tif]

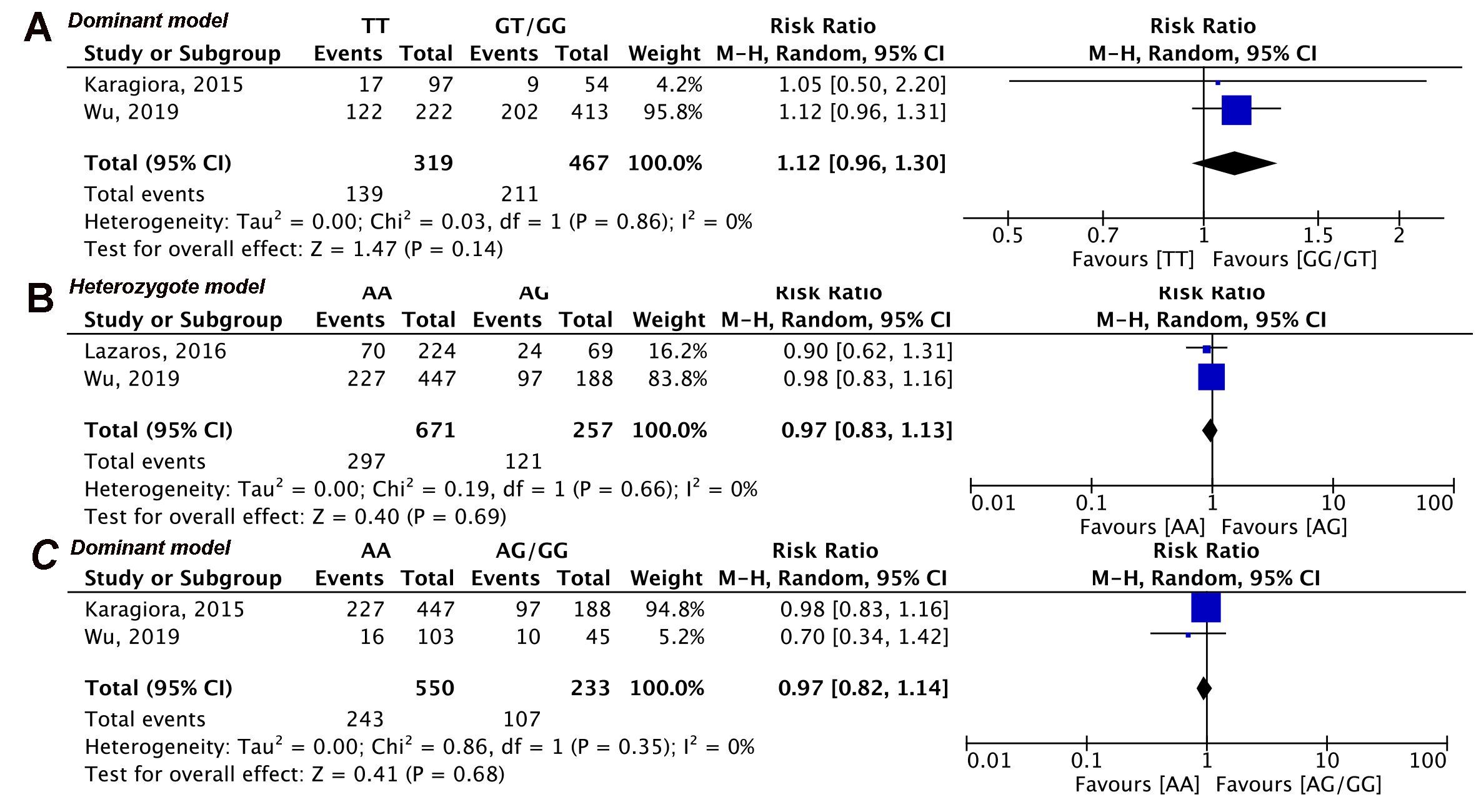

Supplement: Supplementary file 5 — Additional file 5 Supplementary Fig. 5. Forest plots of differences among AMH (rs10407022) and AMHR2 (rs2002555) genotype carriers regarding the pregnancy rate. (A) dominant model of AMH (rs10407022), (B) heterozygote model of AMHR2 (rs2002555), (C) dominant model of AMHR2 (rs2002555). [file 13048_2020_699_MOESM5_ESM.tif]
