# Supplementary material for: Can polymorphisms of AMH/AMHR2 affect ovarian stimulation outcomes? A systematic review and meta-analysis
Source: J Ovarian Res. 2020 Sep 4;13:103. doi: 10.1186/s13048-020-00699-4 (PMC7487641; doi:10.1186/s13048-020-00699-4)
Supplement: Supplementary file 6 — Additional file 6. [file 13048_2020_699_MOESM6_ESM.doc]

**Appendix 1. Search strategy in Pubmed**

#1 AMH (3881)

#2 Anti Mullerian Hormone (4311)

#3 AMHR (43)

#4 AMH receptor (314)

#5 #1 OR #2 OR #3 OR #4 (5305)

#6 polymorphism (320676)

#7 genotype (474591)

#8 genetic (1649012)

#9 pharmacogenetics (18176)

#10 #6 OR #7 OR #8 OR #9 (1868358)

#11 IVF (26079)

#12 in vitro fertilization (46636)

#13 COH (3676)

#14 controlled ovarian stimulation (4968)

#15 controlled ovarian hyperstimulation (2542)

#16 ICSI (10574)

#17 #11 OR 12 OR #13 OR #14 OR #15 OR #16 (66890)

#18 #6 AND #11 AND #17 (97)
